# Role of TNF-α-induced m6A RNA methylation in diseases: a comprehensive review

**DOI:** 10.3389/fcell.2023.1166308

**Published:** 2023-07-24

**Authors:** Youlin Wang, Jing Liu, Yongchen Wang

**Affiliations:** ^1^ Department of Dermatology, The Second Affiliated Hospital of Harbin Medical University, Harbin, Heilongjiang, China; ^2^ General Practice Department, The Second Affiliated Hospital of Harbin Medical University, Harbin, Heilongjiang, China

**Keywords:** N6-methyladenosine (m6A), TNF-α, signaling pathway, immunity, autoimmune disease

## Abstract

Tumor Necrosis Factor-alpha (TNF-α) is ubiquitous in the human body and plays a significant role in various physiological and pathological processes. However, TNF-α-induced diseases remain poorly understood with limited efficacy due to the intricate nature of their mechanisms. N6-methyladenosine (m6A) methylation, a prevalent type of epigenetic modification of mRNA, primarily occurs at the post-transcriptional level and is involved in intranuclear and extranuclear mRNA metabolism. Evidence suggests that m6A methylation participates in TNF-α-induced diseases and signaling pathways associated with TNF-α. This review summarizes the involvement of TNF-α and m6A methylation regulators in various diseases, investigates the impact of m6A methylation on TNF-α-induced diseases, and puts forth potential therapeutic targets for treating TNF-α-induced diseases.

## Introduction

Tumor Necrosis Factor-alpha (TNF-α), a cytokine ubiquitous in the human body, is pivotal in the pathogenesis of various diseases, particularly immune and inflammatory disorders. The intricate mechanisms and aggressive therapeutic interventions necessitate a further investigation of the underlying pathogenesis and potential therapeutic targets. The widespread involvement of m6A methylation in physiopathological processes is a well-established phenomenon. Recent studies have demonstrated the existence of m6A-induced gene expression regulation in various TNF-α-induced diseases. This regulation involves the participation of the writer, eraser, and reader and plays a crucial role in multiple biological processes, including RNA stability, degradation, and variable cleavage. This study aims to summarize the TNF-α-induced diseases and elucidate the relationship between signaling pathways and m6A methylation. This endeavor provides a promising opportunity to gain further insights into the role of m6A methylation in TNF-α-induced diseases and identify potential therapeutic targets.

### TNF-α

TNF-α, or cachectin and TNFSF1A, is a constituent of the tumor necrosis factor superfamily and comprises a 157 amino acid homotrimeric protein (Campanati A et al., 2019). Various immune cells, including macrophages, monocytes, neutrophils, CD4^+^ T cells, and Natural killer cells (NK cells), secrete TNF-α in response to endotoxin stimulation (Chadwick et al., 2008). TNF-α exits in two biologically active forms: transmembrane TNF-alpha (tmTNF-α) and secretory TNF-alpha (sTNF-α). The former predominantly exists in a membrane-bound configuration and is converted into sTNF-α by the TNF-α converting enzyme TACE. The systemic circulation is the primary site of action for sTNF-α, imparting an endocrine role to TNF-α (Yang et al., 2018).

The cellular receptors for TNF-α are classified into TNFR1 and TNFR2. TNFR1 is expressed ubiquitously on almost all cells and can be activated by sTNF-α and tmTNF-α. It interacts with TNFR1-associated death domain (TRADD) adaptor proteins (Pobezinskaya and Liu, 2012). Conversely, TNFR2 expression is restricted to immune cells, endothelial cells, and neurons. Furthermore, sTNF signaling via TNFR1 primarily initiates pro-inflammatory pathways, while tmTNF binds to TNFR2 for immune regulation and tissue regeneration (Zhang et al., 2017).

### Role of TNF-α in autoimmune/inflammatory diseases

**FIGURE 1:**
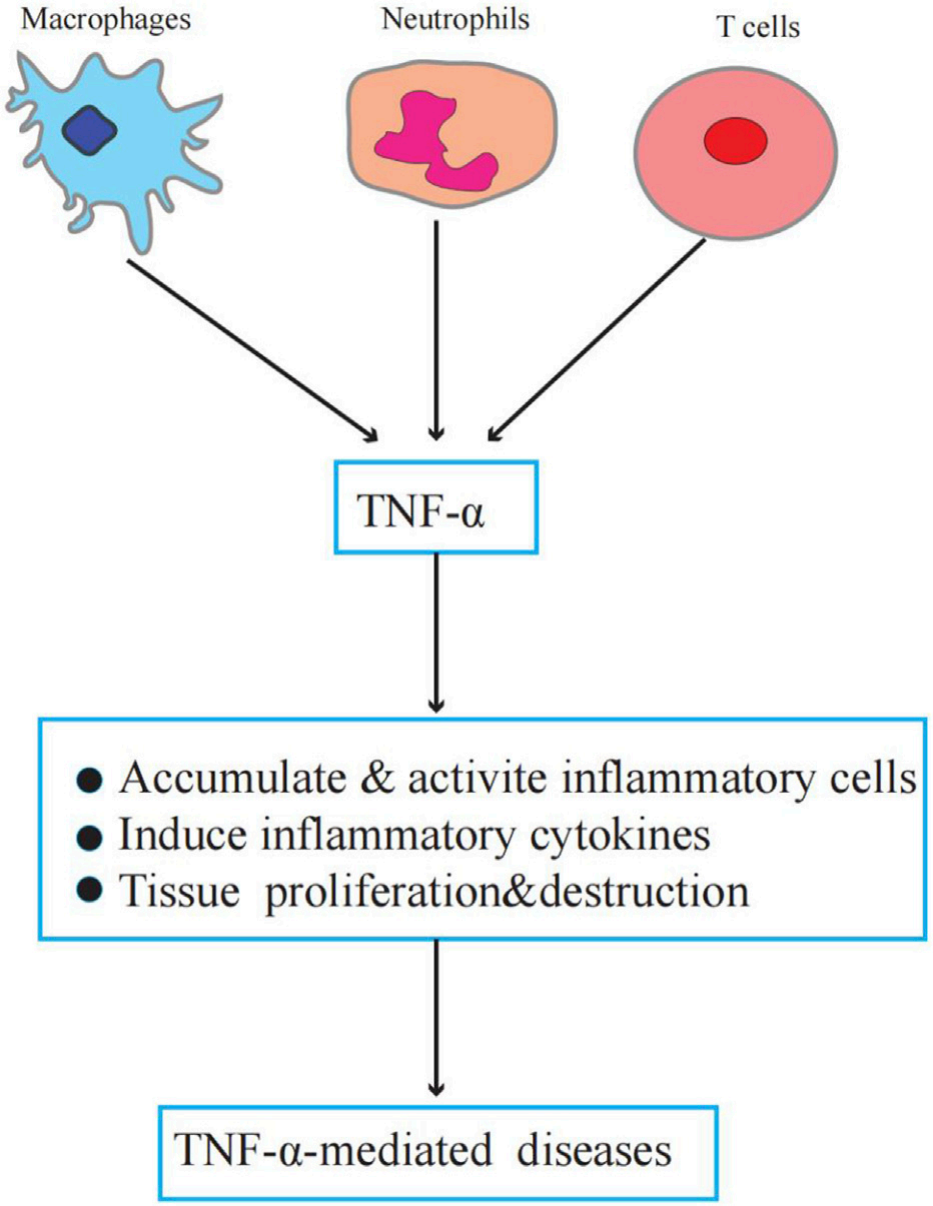
The role of TNF-α in TNF-α-induced diseases. Macrophages, neutrophils, and T cells secrete TNF-α. TNF-α promotes inflammatory cell activation and accumulation induces inflammatory factor production, forms a local inflammatory environment, and leads to tissue proliferation and destruction.

TNF-α plays an important role in physiology and pathology. Physiologically, TNF-α is a critical component of normal immune responses. TNF-α is regulated by immune system activation and has various biological activities, such as regulating inflammation, apoptosis, chemokine production, and metabolism (Brenner et al., 2015). However, abnormal or excessive TNF-α activation can lead to or aggravate chronic inflammation and neoplastic diseases (Idriss and Naismith, 2000; van Horssen et al., 2006). Figure 1 illustrates the role of TNF-α in TNF-α-induced diseases.

Autoimmune/inflammatory diseases are a series of tissue injuries caused by systemic or organ-specific inflammation (Wang L et al., 2015). Epidemiology suggests that autoimmune/inflammatory diseases affect 3%–5% of the population (Surace and Hedrich, 2019). Autoimmune/inflammatory diseases are monogenic or polygenic, with autoantibodies and/or reactive lymphocyte populations being induced. They are influenced by individuals and the environment, ultimately leading to disease development (Wang X et al., 2015; He et al., 2018). TNF-α is widely involved in autoimmune/inflammatory diseases, such as rheumatoid arthritis (RA), inflammatory bowel disease (IBD), ankylosing spondylitis (AS) arteriosclerosis, psoriasis (PS), and noninfectious uveitis (NIU). TNF-α expression is elevated in tissues and/or blood and is associated with disease activity in TNF-α-induced autoimmune/inflammatory diseases (Arican et al., 2005; Wehkamp et al., 2016; Bullock et al., 2018; Ebrahimiadib et al., 2021). Meanwhile, TNF-α inhibitors have proven therapeutic in treating the above diseases (Udalova et al., 2016). These studies suggest an important role for TNF-α in autoimmune/inflammatory diseases.

Macrophages and Th1 cells secrete TNF-α in RA, activate synovial fibroblasts, and produce excessive cathepsins and MMPs, destroying cartilage and bone and causing joint erosion. TNF-α promotes osteoclast generation and induces synovial hyperplasia and angiogenesis. TNF-α can also produce other inflammatory cytokines and promote an inflammatory milieu in the synovium, manifesting clinically as pain and joint swelling (Koch et al., 1994; Brennan and McInnes, 2008; Lin et al., 2020).

Stressed keratinocytes secret TNF-α and other cytokines in PS, activating DCs macrophages and neutrophil accumulation, which is pathologically characterized by parakeratosis, acanthosis, and Munro microabscesses. TNF-α also induces keratinocyte proliferation and anti-apoptosis via the NF-κB/MAPK signaling pathway (Jang et al., 2021).

IBD primarily comprises Crohn’s disease (CD) and ulcerative colitis (UC). TNF-α induces pro-inflammatory effects, including macrophages and T cell activation, chemotaxis of neutrophils and monocytes, increased endothelial cell adhesion molecules (EcAMs) expression, and activation of coagulation and fibrinolytic responses, resulting in epithelial injury, endothelial activation, and vascular destruction. TNF-α also promotes intestinal fibrosis caused by intestinal fibroblasts, leading to intestinal strictures formation (Liou and Storz, 2010; Bounder et al., 2020).

### Role of TNF-α in tumors

TNF-α is a double-edged sword in cancer. When TNF-α is secreted in excess, it stimulates tumor cells to release pro-inflammatory mediators, inhibits the anti-tumor activity of immune cells, suppresses mucosal healing, and disrupts mucosal barrier function, inducing cancer cell proliferation, immune escape, invasion, angiogenesis, and metastasis (Havell et al., 1988). The mechanisms may be related to reactive oxygen species (ROS) and reactive nitrogen species (RNS) generation (Adegbola et al., 2018). High TNF-α concentrations may also play an anti-tumor role (Levin et al., 2016). Additionally, the role of TNF-α depends on its expression site in the tumor (Landskron et al., 2014).

### m6A methylation

m6A refers to the adenine methylation at position N6 and is a rich and extensive RNA modification, accounting for approximately 80% of all RNA methylation modifications (Dominissini et al., 2012; Yue et al., 2015). m6A modifications are widespread in mammals, plants, and viruses and are highly conserved among species (Horowitz et al., 1984; Fu et al., 2014; Liu et al., 2014).

Mammalian m6A methylation sites near the mRNA stop codon and in the 3′untranslated region (3′UTR) (Fu et al., 2014), non-coding RNAs, such as long non-coding RNAs (lncRNAs) and microRNAs (miRNA), also have m6A modification sites (Alarcón et al., 2015a; Jacob et al., 2017). m6A transferases, demethylases, and binding proteins regulate involvement in m6A modification and play essential roles in biological processes, such as mRNA stability, degradation, alternative splicing, nuclear export, and translation (Wang et al., 2014; Zaccara et al., 2019; Wei and He, 2021). A recent study of m6A methylation has demonstrated a rapid increase trend with the rapid update of new technologies. m6A modification plays an essential role in the body’s primary metabolism and basic physiological functions (Dominissini et al., 2012; Frye et al., 2018; Shi et al., 2019). Methylation of m6A has been studied in various diseases, such as tumors, cardiovascular diseases, neurological diseases, viral infections, and autoimmune inflammation (Zhao et al., 2014; Geula et al., 2015; Sun et al., 2019). The m6A binding protein binds to the corresponding m6A modification site to achieve their respective biological functions and can also change RNA secondary structure and play a regulatory role (Zhang C et al., 2019; Chai et al., 2021).

Enzymes involved in catalyzing and recognizing m6A modifications can be divided into writer, eraser, and reader based on their function. Table 1 presents their functions. The writer, including major components, such as methyltransferase-like 3 (METTL3), methyltransferase-like14 (METTL14), and Wilms’ tumor 1-associated protein (WTAP), is primarily responsible for catalyzing m6A modifications and writing methyl groups in RNA. METTL3 is a prominent constituent member of this complex. METTL3 localizes to nuclear patch areas of the nucleus and forms a stable heterodimer with METTL14 (Liu et al., 2020; Zeng et al., 2020). METTL3 recognizes S-adenosyl methionine (SAM) during catalysis. It exhibits catalytic activity (Liu et al., 2014), whereas METTL14 recognizes RNA substrates and methyl localization, enhancing methyltransferase activity besides stabilizing METTL3 (Wang et al., 2016; Zhang et al., 2020). WTAP regulates the METTL3/14 accumulation on mRNAs and the specific mRNA methylation as its primary function (Fan et al., 2022). Other components of the m6A methyltransferase complex include KIAA1429, RNA-binding motif protein 15 (RBM15), and Zinc finger CCCH-type containing 13 (ZC3H13) (Hu et al., 2016; Huang et al., 2021; Jiang et al., 2021; Zhang, et al., 2022a).

**TABLE 1:** Writers, erasers, and readers for m6A.

	Name	Function
Writer	METTL3	Catalytic production of m6A modification
	METTL14	1. Enhance methyl transferase activity in addition to stabilizing METTL3
		2. Recognize RNA substrates and methyl localization
	WTAP	1. Regulate METTL3/14 accumulation toward mRNAs
		2. Methylation of specific mRNAs
Eraser	FTO	Demethylation
	ALKBH5	1. Demethylation
		2. Regulates mRNA export and stability
Reader		
nucleus	YTHDF2	Facilitates degradation
	YTHDC1	Alternative splicing, alternative polyadenylation, and nuclear export
	(HNRNP) A2B1/C/G	Pre-mRNA processing, mRNA metabolism, and nuclear export
cytoplasm	YTHDF1/3, eIF	Facilitate translation
	YTHDC2	mRNA stability and translation
	(IGF2BP) 1/2/3	mRNA stability and translation

m6A demethylase catalyzes the reversible m6A modification process. Demethylases, primarily Fat mass and obesity-associated protein (FTO) and Alkylation repair homolog protein 5 (ALKBH5), play a role in catalyzing demethylation (Ito et al., 2011). FTO belongs to the iron/α-ketoglutarate–dependent AlkB protein family and is tightly linked to obesity (Dina et al., 2007; Frayling et al., 2007; Gerken et al., 2007). However, the substrate-bound by FTO does not appear to be m6A alone. It has been claimed that FTO impacts mRNA stability and translation efficiency by regulating m6A.m. levels (Mauer et al., 2017). Moreover, FTO localization varies in different cells, with FTO mediating the m6A demethylation in the nucleus and mediating the demethylation of m6A.m. and m6A in the cytoplasm (Wei et al., 2018). Except for demethylation, ALKBH5 regulates mRNA export and stability by affecting the splicing and mRNA production rates and participating in functions, such as mRNA transport, metabolic processing, and degradation (Zheng et al., 2013; Li et al., 2020).

The “writer” and “eraser” of m6A act on intranuclear mRNAs, making m6A modifications dynamic and reversible. Furthermore, downstream regulation at the mRNA translation level by the “reader” is required to achieve biological function. Some m6A readers have biological roles in the nucleus. Nuclear m6A readers include nuclear ribonucleoprotein (HNRNP) A2B1/C/G, YT521-B homology (YTH) domain-containing heterogeneous proteins, such as YTHDC1 (Zhang et al., 2021). HNRNPs are RNA-binding proteins associated with the precursor mRNA (pre-mRNA) maturation in the nucleus and affect processes, such as pre-mRNA processing, mRNA metabolism, and nuclear export (Das, et al., 2019). HNRNPA2B1 initially promotes the primary miRNA processing and maturation by binding transcription of m6A methylation (Alarcon et al., 2015b). YTHDC1 mediates alternative splicing, alternative polyadenylation, and nuclear export (Xiao et al., 2016; Chen et al., 2021). A portion of the reader plays a biological role in the cytoplasm. Extranuclear m6A reader primarily includes YTHDF1/2/3, YTHDC2, and Insulin-like growth factor 2 mRNA binding protein (IGF2BP) 1/2/3 (Xiao et al., 2016). YTHDF1/3 and eIF can act on mRNA translation initiation factors and contribute to mRNA translation (WANG X et al., 2015). YTHDF2 binds to mRNA and promotes its degradation, affecting stability and translation status (Wang and Liu, 2021). IGF2BP1/2/3 promotes m6A-induced mRNA stability and translation (Liu et al., 2015).

### TNF-α and m6A methylation

**FIGURE 2:**
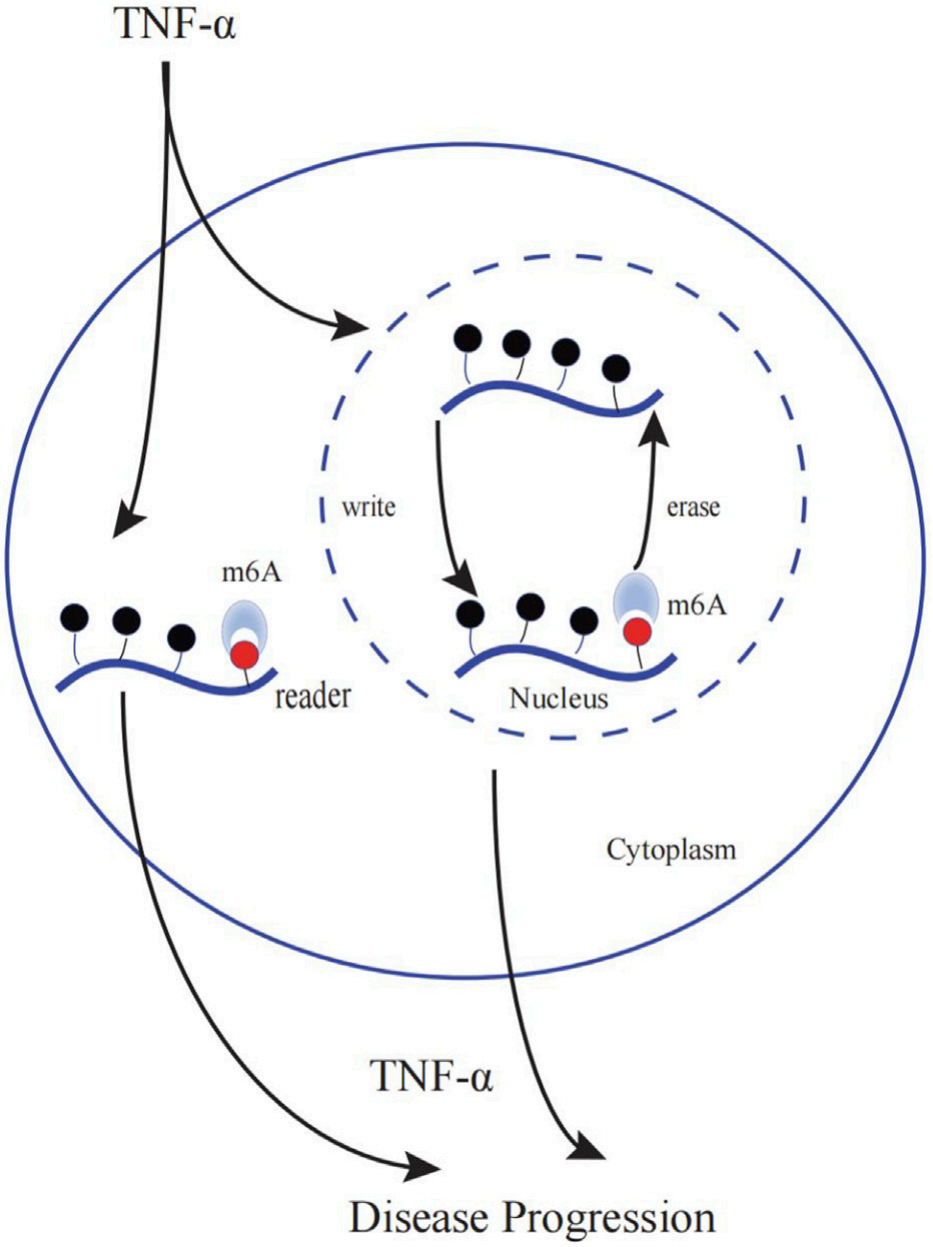
The role of m6A in TNF-α-medicated diseases. m6A regulator levels (writers, erasers, and readers) change upon TNF-α stimulation. Simultaneously, TNF-α levels change with altered m6A regulator expressions, implying that TNF-α and m6A regulators interact with each other and jointly impact the disease condition. However, the causal relationship between TNF-α and m6A regulators is currently unknown.

Many studies have revealed that m6A methylation is involved in TNF-α-induced disease progression. It mainly involves changes in global m6A methylation, enzyme levels and activities, and TNF-α-induced pathways. Figure 2 shows the role of m6A in TNF-α-medicated diseases.

Li et al. (2021) discovered that differentially expressed m6A methylation sites between lncRNAs and circRNAs were primarily enriched in TNF signaling pathways after mapping m6A methylation profiles in bladder cancer. These results suggest that TNF signaling may differ in global m6A methylation levels in some diseases.

m6A regulator levels (writers, erasers, and readers) change upon TNF-α stimulation, ultimately impacting the disease. These results suggest that m6A methylation regulators may be involved in TNF-α-induced disease progression. Wang, et al. (2019) discovered that TNF-α stimulation decreased FTO expression in mesenchymal stromal cells (MSCs), shortened the half-life of Nanog mRNA, led to decreased mRNA and protein expression, and ultimately significantly inhibited the ability of MSCs to differentiate into sweat gland cells. Jian D et al. (2020) discovered that when endothelial cells are stimulated with TNF-α, METTL14 expression increases, acts directly on the promoter regions of adhesion molecule-1 (VCAM-1) and intercellular adhesion molecule 1 (ICAM1), increases the FOXO1 gene m6A methylation level and promotes its transcription, enhances expression, ultimately leads to atherosclerotic plaque formation. METTL14 binds to the engulfment and cell motility 1 (ELMO1) mRNA 3 ‘UTR and promotes mRNA degeneration in Ankylosing spondylitis. TNF-α stimulation decreased METTL14 expression and decreased the ELMO1 degeneration rate, thereby increasing ELMO1 expression, leading to enhanced directional migration of AS-MSC *in vivo* and *in vitro*, ultimately aggravating the disease (Xie et al., 2021). Zhu et al. (2021) revealed that METTL14 expression positively correlated with TNF-α concentration in abnormal nucleus pulposus cells. METTL14 was involved in TNF-α-induced m6A modification of miR-34a-5p and interacted with DGCR8 to promote cell cycle arrest and aberrant degeneration in nucleus pulposus (NP) cells from intervertebral disc degeneration (IVDD) patients. In lumbar disc herniation (LDH), WTAP can bind to METTL3 and METTL14 as well as NORAD to promote its m6A methylation. Following TNF-α stimulation of nucleus pulposus cells, WTAP expression was decreased.RNA immunoprecipitation (RIP) revealed that YTHDF2 was the predominant reader protein, promoting NORAD degradation and ultimately exacerbating disease (Li et al., 2022).

Altered m6A methylation regulator levels can also significantly affect TNF-α levels and/or related signaling pathway expressions, ultimately affecting the disease. Li et al. (2020) indicated that m6A RNA modification is essential for Th cell differentiation and proliferation *in vivo*. METTL3 knockdown mice significantly decreased the proportion of naive T cells differentiating into Th1 and Th17 cells and a significant increase in Th2 cells. METTL14 expression is significantly increased in diabetic nephropathy, and METTL14 overexpression leads to increased inflammatory factor secretion, such as TNF-α in serum. Mechanistically, METTL14 downregulated α-klotho expression in an m6A-dependent manner, leading to glomerular endothelial cell apoptosis in the kidneys of diabetic nephropathy (DN) patients (Li M.et al., 2021). Sang et al. (2021) exposed that METTL3 expression was decreased in osteoarthritis (OA), and METTL3 overexpression decreased inflammatory factor levels, such as TNF-α. METTL3 overexpression adjusts to the balance between TIMPs and MMPs, possibly leading to extracellular matrix degradation in OA. ALKBH5 expression has been decreased in TNF-α-treated human umbilical vein endothelial cells (HUVECs). ALKBH5 inhibits TNF-α-induced endothelial cell apoptosis via Bcl-2, thereby reducing atherosclerosis (Zhang T. et al., 2022). Studies have revealed that METTL3 is overexpressed in acute kidney injury (AKI) models, and METTL3 deficiency reduces renal cell inflammation and programmed cell death induced by TNF-α. METTL3 promoted m6A modifications of TAB3 and enhanced stability via IGF2BP2-dependent mechanisms (Wang B et al., 2022). METTL3 expression is reduced in temporomandibular joint osteoarthritis (TMJ OA). Further investigation indicated that TNF-α-induced osteoclast autophagy and apoptosis are inhibited via the m6A/Ythdf1/Bcl2 signaling axis (He et al., 2022). METTL3 expression was increased in mouse fungal keratitis (FK). METTL3 deficiency reduced TNF-α expression and decreased PI3K/AKT pathway protein expression, ultimately inhibiting the inflammatory response (Huang et al., 2022). FTO expression was reduced in LPS-treated cardiomyocytes, and FTO knockdown in cardiomyocytes may lead to inflammatory cytokine overexpression, such as TNF-α. The m6A-RNA immunoprecipitation (MeRIP) assay revealed that FTO knockdown would lead to TNF-α mRNA transcript hypermethylation (Dubey et al., 2022). YTHDF1 was downregulated and acts as YTHDF1/WWP1/NLRP3/caspase-1 axis in sepsis. Overexpressed YTHDF1 stabilizes the TNF-α expression in CLP-induced mice (Zhang et al., 2022c). The YTHDF2 expression was increased in lipopolysaccharide (LPS)-induced RAW 264.7 cells, and the YTHDF2 knockdown significantly increased the TNF-α, MAP2K4, MAP4K4, and activated downstream signaling pathway expressions, such as NF-κB and MAPK (Yu H et al., 2019). However, YTHDF2 expression was elevated in LPS-induced osteoclastogenesis cells, and the YTHDF2 knockdown significantly increased the inflammatory factor expression levels, such as TNF-α and activated downstream pathways, including NF-κB and MAPK signaling (Fang et al., 2021).

Influencing the activity of m6A methylation regulators can similarly influence TNF-α levels and disease progression. LuHui derivative (LHD), an FTO inhibitor, binds explicitly the pocket containing the regulatory sites of RNA methylation in LHD protein, leads to loss of FTO function, inhibits palmitate-induced cardiac inflammation, reduces CD36 expression by reducing the stability of CD36 mRNA, and inhibits inflammatory factor expressions, such as TNF-α and IL-6, thereby relieving hyperlipidemic cardiomyopathy (Yu et al., 2021). Emodin decreases inflammation, such as TNF-α expression in LPS-treated 1321N1 cells, by inactivating METTL3-induced NLRP3 expression (Wang J et al., 2022). Further, the important role of m6A methylation in TNF-α-induced diseases is illustrated.

In summary, m6A methylation is widely involved in TNF-α-induced diseases involving various diseases and cells. Table 2 presents the role of m6A methylation in TNF-α-medicated diseases. However, whether TNF-α acts directly on m6A methylation regulators and how m6A methylation regulators are altered in TNF-α-induced diseases still needs further investigation.

**TABLE 2:** m6A in TNF-α-medicated diseases.

Disease/cells	m6A	Up/Downstream	Functional outcome/correlation
	Regulator (s)	Target (s)	
MSCs	FTO↓	Nanog↓	Reduce stability
Atherosclerosis	METTL14↓	FOXO1↑	Promote transcription
Ankylosing spondylitis	METTL14↓	ELMO1↑	Reduce denaturation rate
			Enhance directional migration
Intervertebral disc degeneration	METTL14↑	MiR-34a-5p↑	Cell cycle arrest and senescence
Lumbar disc herniation	WTAP↓	NORAD↓	Facilitates degradation
Diabetic nephropathy	METTL14↑	α-klotho↓	Decrease mRNA stability
Osteoarthritis	METTL3↓	MMP1↑, MMP3↑	Matrix degradation
		MMP13↓, TIMP-1↓, TIMP-2↓	
Atherosclerosis	ALKBH5↓	Bcl-2↓	Apoptosis
Nephritis	METTL3↑	TAB3↑	Enhance stability
Temporomandibular joint osteoarthritis	METTL3↓	m6A/Ythdf1/Bcl2 axis↓	Enhance stability (Ythdf1)
	YTHDF1↓		
Fungal keratitis	METTL3↓	PI3K/AKT pathway↑	Unknown
Myocardial inflammation	FTO↓	Unknown	Unknown
Sepsis	YTHDF1↓	YTHDF1/WWP1/NLRP3/caspase-1 axis↑	Promote transcription
RAW 264.7 cells	YTHDF2↑	MAP2K4, MAP4K4↑	Stabilization
Osteoclastogenesis	YTHDF2↑	NF-κB and MAPK pathway↑	Reduce stabilization

↑: upregulate ↓: downregulate.

### TNF-α signaling pathways

TNF-α exerts its biological function primarily via nuclear factor-kappaB (NF-κB) and mitogen-activated protein kinases (MAPK) signaling pathways. Studies have revealed that the above signaling pathways are also involved in m6A methylation in other diseases.

NF-κB is a eukaryotic transcription factor family with five subunits: p65 (RelA), RelB, c-Rel, p105/p50, and p100/p52. It involves various processes, such as immune response, inflammation, apoptosis, growth, and development (Lawrence, 2009; Wertz and Dixit, 2010; Kumar et al., 2015). NF-κB signaling pathway contains canonical and non-canonical signaling pathways. Classical signaling pathways initiate transcription by degrading IκB and releasing NF-κB dimers, translocating to the nucleus and activating target genes rapidly but transiently (Chen and Greene, 2004; Ruland, 2011). A few TNF superfamily receptors activate non-canonical signaling pathways. The central event of the non-canonical NF-κB signaling pathway is processed for p100 to obtain p52. Proteolytic processing of p100 leads to the p52 production and RelB release, forming a RelB/p52 dimer and translocation to the nucleus, thereby activating signaling pathways (Dejardin, 2006; Hayden and Ghosh, 2008; Sun, 2011).

The m6A methylation is involved in the NF-κB pathway in various diseases and impacts the disease. METTL3 has been overexpressed in RA, positively associated with disease activity. Furthermore, METTL3 inhibited LPS-induced macrophages in inflammation via NF-κB (Wang et al., 2019). Specific METTL14 deletion leads to colon stem cell apoptosis in IBD, mucosal barrier dysfunction, and severe colitis. Mechanistically, METTL14 mitigates colonic epithelial cell death by controlling Nfkbia mRNA stability and the NF-κB signaling pathway (Zhang et al., 2022d). Mice with loss of METTL3 in myeloid cells develop less age-associated incidence of non-alcoholic fatty liver disease (NAFLD) and obesity. In macrophages, METTL3 deficiency may cause significantly increased DNA Damage Inducible Transcript 4 (DDIT4) expression and activates the mTORC1/NF-κB signaling pathway, finally improving NAFLD (Qin et al., 2021). The research discovered that IL-6 cooperates with CUDR, causing METTL3 to interact with SUV39h1 mRNA3 ‘UTR and promote SUV39h1 expression. SUV39h1 enhances the NF-κB expression and phosphorylation, leading to malignant transformation of the human embryonic hepatocyte-like stem cells (Zheng Q et al., 2016). ALKBH5 had higher expression in cancer stem cells than in normal tissue. TLR4 increases ALKBH5 expression via activating the NF-κB pathway. The upregulated ALKBH5, whose m6A modification gene is NANOG, promotes ovarian cancer cell aggressiveness (Jiang et al., 2020).

MAPK is a signal-regulated protein kinase important in cell proliferation, differentiation, autophagy, apoptosis, immune response, and other physiological and pathological conditions (Arthur and Ley, 2013). MAPKs act simultaneously as signaling messengers, transducing extracellular signals into the cell and regulating the various cytokine and chemokine expressions (Zhao et al., 2018). MAPK signaling pathway comprises four subfamilies: ERK1/2, JNK1/2/3, p38 MAPK, and ERK5 (Uddin et al., 2021). This signaling pathway activates the MAPKs in a three-tier kinase activity from MAPKKK, MAPKK to MAPK (Burotto et al., 2014). The m6A methylation is widely involved in regulating the MAPK signaling pathway. METTL3 expression is decreased in tumor-infiltrating NK cells. SHP-2, an m6A-modified gene whose protein expression is reduced in METTL3-deficient NK cells, may render NK cells hyporesponsive to IL-15 via the MAPK signaling pathway (Song et al., 2021). YTHDF2 interacts with mRNAs encoding proteins, epithelial-to-mesenchymal transition, and increased translation rates in MYC-driven breast cancer to inhibit cell death via the MAPK signaling pathway (Einstein et al., 2021). Highly expressed WTAP is associated with shorter overall survival and lymph node metastasis in ovarian cancer cells. WTAP downregulation decreased several MAPK protein expressions, such as p-ERK, ERK, p-JNK, and JNK, suggesting a possible correlation between WTAP and MAPK signaling pathways (Yu R et al., 2019). METTL3, whose m6A target modification is miR-1246, is upregulated in colorectal cancer and promotes cell migration and invasion. SPRED2 is a downstream target of miR-1246, and its downregulation promotes MAPK signaling pathway expression (Peng et al., 2019).

## Conclusion and future perspectives

This study reviews TNF-α and TNF-α-induced diseases and summarizes the role of m6A methylation in TNF-α-induced diseases and the main signaling pathways involved in TNF-α. Moreover, an in-depth study of m6A methylation may elucidate the pathogenesis of TNF-α-induced diseases and provide more possibilities to identify potential therapeutic targets.

If an m6A methylation regulator is aberrantly expressed and changes with TNF-α levels in TNF-α-induced diseases, we can search the binding sites of m6A methylation regulators to differentially expressed genes via methylated RNA immunoprecipitation sequencing (MeRIP-seq) and transcriptomic RNA sequencing (RNA-seq).

Studies have demonstrated that the external environment influences the m6A methylation level. Cigarette smoke condensate (CSC) induces hypomethylation of the METTL3 promoter, resulting in aberrant METTL3 overexpression. The miR-25–3p promotes the pancreatic ductal adenocarcinoma (PDAC) development in smokers by inhibiting PH domain leucine-rich repeat protein phosphatase 2 (PHLPP2) and activating AKT-p70S6K (Zhang J et al., 2019). Chronic exposure of human bronchial epithelial (HBE) cells to arsenite sodium (NaAsO_2_) increases m6A methylation levels significantly and induces a malignant phenotype. Silencing METTL3 reversed these changes (Gu et al., 2018). Given the potential link between TNF-α and m6A methylation, whether TNF-α affects RNA functions, such as secondary structure, decoration affinities, and protein binding partners, by affecting m6A methylation regulators remains to be investigated.

Thus, m6A modification is a complex process working as a double-edged sword for TNF-α-medicated diseases. Does the relationship between TNF-α and m6A methylation regulators are causal or associations? Therefore, unknown regulators exist between TNF-α and m6A methylation. Furthermore, TNF-α has a dual role of cytokines and inflammatory mediators. It is also worth investigating whether TNF-α and m6A methylation regulators can influence each other between different cells. For example, in TNF-α-induced diseases that concern various immune cells, can changes in the m6A methylation regulator expression levels in one cell influence the m6A methylation regulator levels and TNF-α in another immune cell? Further exploration is needed.

Modulators of m6A methylation provide novel potential therapeutic targets. If altered levels of m6A methylation regulators improve disease conditions, inhibitors/activators targeting m6A methylation regulators may provide potential therapeutic possibilities. The newly identified m6A modifiers will open novel therapeutic avenues to understand the m6A methylation-associated diseases.
